# Clinical Evaluation of Long-Read Sequencing for Telomere Length Assessment in Human Blood and Lung Tissues

**DOI:** 10.3390/cells15131165

**Published:** 2026-06-26

**Authors:** Viviana P. Lutzky, Subash K. Rai, Maxine E. Tan, Penny L. Groves, Kiara M. Knuckey, John A. Mackintosh, Mathew J. K. Jones, Simon H. Apte, Daniel C. Chambers

**Affiliations:** 1 Queensland Lung Transplant Service, The Prince Charles Hospital, Brisbane, QLD 4032, Australia; 2 School of Medicine, Faculty of Health, Medicine and Behavioural Sciences, The University of Queensland, Brisbane, QLD 4072, Australia; 3 Australian Centre for Cellular Ageing, The Prince Charles Hospital, Brisbane, QLD 4032, Australia; 4 Frazer Institute, Faculty of Health, Medicine and Behavioural Sciences, The University of Queensland, Brisbane, QLD 4102, Australia; 5 School of Chemistry & Molecular Biosciences, Faculty of Science, University of Queensland, Brisbane, QLD 4072, Australia

**Keywords:** telomere length, Flow-FISH, long-read sequencing, Telo-Seq, pulmonary fibrosis, telomere biology disorders

## Abstract

**Highlights:**

**What are the main findings?**

- Telomere length measured by long-read sequencing (Telo-Seq) correlated with Flow-FISH (R^2^ ≈ 0.77) and exhibited lower assay variability (CV ~1.4% compared to ~4.6%).
- Telo-Seq and Flow-FISH showed largely concordant classification.
- Telo-Seq enabled exploratory telomere length assessment across multiple biospecimens, including PBMC, whole blood, saliva, whole lung lavage fluid, and lung tissue.

**What are the implications of the main findings?**

- Long-read sequencing offers a reproducible, high-resolution approach to telomere length measurement, supporting its use as a complementary clinical assay.
- Long-read sequencing may detect biologically relevant telomere shortening not captured by population-averaged assays. Whether this improves clinical diagnosis or risk stratification requires validation in larger cohorts with clinical outcome data.
- The ability of long-read sequencing to measure telomere length in disease-relevant tissues may improve understanding of tissue-specific telomere biology in pulmonary fibrosis and related disorders.

**Abstract:**

Telomere length (TL) measurement is increasingly used in the evaluation of pulmonary fibrosis and suspected telomere biology disorders, with flow cytometry–fluorescence in situ hybridisation (Flow-FISH) representing the current clinical reference standard. However, Flow-FISH provides a population-averaged relative TL, has a high analytical variability and is largely restricted to PBMC-based measurements. We evaluated a long-read sequencing–based assay, Telo-Seq, for clinical TL assessment in an Australian cohort. In this observational study, TL was measured by both Flow-FISH and Telo-Seq in PBMC from 156 healthy controls and a small cohort of 24 patients undergoing clinical evaluation for interstitial lung disease, using the same blood processing and assay workflows. In an exploratory subset of samples, TL was also assessed in whole lung lavage fluid and explanted lung tissue. In the assessment of PBMC, Telo-Seq correlated with Flow-FISH (R^2^ ≈ 0.77) and showed lower inter-assay variability (CV ~1.4% vs. ~4.6%). Classification relative to assay-specific age-adjusted 10th-percentile thresholds was largely concordant between Flow-FISH and Telo-Seq, although discordant cases occurred in both directions near clinically relevant thresholds. Long-read sequencing provides a reproducible approach to TL assessment and supports further evaluation in disease-relevant tissues.

## 1. Introduction

Telomeres are hexanucleotide (TTAGGG) repeats that cap chromosome ends and preserve genomic stability by preventing end-to-end fusions and replicative senescence. Progressive telomere shortening and defects in telomere-maintenance pathways, including variants in genes such as *TERT*, *TERC*, and *RTEL1*, underlie a spectrum of age-related and regenerative-failure disorders (reviewed in ref. [1]). Pulmonary fibrosis (PF) is associated with telomere shortening and represents the major clinical context in which telomere length (TL) assessment has diagnostic, prognostic, and therapeutic relevance (reviewed in ref. [2]).

Approximately one-third of familial PF kindreds harbour pathogenic variants in telomere-maintenance genes, and up to half of patients with apparently sporadic PF demonstrate shortened leukocyte telomeres (reviewed in ref. [3]). Clinically, short telomeres are associated with earlier disease onset, extrapulmonary manifestations (including bone-marrow failure and liver disease), reduced survival, and increased post-transplant complications, driving growing interest in accurate TL measurement in peripheral blood [4]. To date, flow cytometry-fluorescence in situ hybridisation (Flow-FISH) has served as the clinical reference standard; however, it reports mean telomere signal, cannot resolve chromosome arm-specific telomeres, and provides limited insight into telomere length distribution or critically short telomeres [5]. This limitation is biologically important because telomere dysfunction may be driven by critically short telomeres within the overall telomere length distribution rather than by mean telomere length alone. Interestingly, a study by Hemann et al. showed that the shortest telomeres, rather than average telomere length, determine chromosome stability and cellular viability [6], while TeSLA-based studies further demonstrate the value of measuring short-telomere distributions rather than relying only on average TL [7]. These observations support the rationale for sequencing-based approaches that can resolve telomere heterogeneity and chromosome-specific telomere lengths.

Recent advances in long-read sequencing have enabled direct telomere measurement with increasing resolution. Early nanopore-based telomere-profiling studies demonstrated that telomere length distributions could be resolved at chromosome-specific resolution in yeast and human cells [8]. Subsequent work applying nanopore telomere-profiling to human blood showed that certain chromosome ends maintain reproducibly longer or shorter telomeres across individuals [9]. Most recently, Schmidt et al. reported a long-read sequencing workflow capable of resolving bulk, chromosome-arm and allele-specific telomere length in human cells [10]. Together, these studies established the technical feasibility and biological value of long-read telomere sequencing. However, most prior work has focused on method development, reference biology, or proof-of-principle applications rather than direct clinical benchmarking against the current reference assay in a disease-specific cohort. As a result, the clinical performance and translational utility of long-read telomere sequencing relative to Flow-FISH in pulmonary fibrosis and suspected telomere biology disorders remain incompletely defined. This represents an important unmet need because accurate telomere length assessment may inform diagnosis, genetic counselling, family screening, transplant risk stratification and treatment planning in patients with pulmonary fibrosis. In particular, it remains unclear whether sequencing-based approaches can identify clinically relevant telomere shortening beyond that detected by existing clinical assays, and whether they can be reliably applied across disease-relevant tissue compartments beyond peripheral blood.

Clinical implementation of telomere testing requires consideration of practical barriers beyond analytical performance, including sample logistics, scalability, turnaround time, cost and inter-laboratory standardisation. Although Flow-FISH remains the current clinical reference standard, testing is typically centralised and requires coordinated handling and transport of viable blood or PBMC samples, which may limit accessibility across geographically dispersed clinical services. Long-read sequencing approaches offer additional resolution, including telomere length distributions and chromosome-end–specific information, and DNA-based workflows may offer future logistical advantages through sample batching and multiplexing. However, clinical adoption of Telo-Seq will require assay-specific reference ranges, harmonised quality-control thresholds, reproducible bioinformatics pipelines, multicentre validation, and formal assessment of cost, scalability, and turnaround time relative to established Flow-FISH testing.

Here, we benchmark Telo-Seq against Flow-FISH in an Australian cohort, assessing concordance between methods, analytical variability and classification relative to age-adjusted telomere length thresholds. We further evaluate a decentralised clinical workflow and explore the feasibility of applying Telo-Seq across paired blood- and lung-derived biospecimens to assess its potential utility for evaluating telomere biology in disease-relevant tissue compartments. Our findings demonstrate strong agreement between the assays while highlighting the potential of Telo-Seq to identify additional patients below assay-specific short-telomere thresholds and enable telomere length measurement across multiple tissue types, supporting its role as a complementary approach for the evaluation of telomere biology in pulmonary fibrosis.

## 2. Materials and Methods

### 2.1. Ethics Statement

The study was conducted in accordance with the Declaration of Helsinki and approved by The Prince Charles Hospital Human Research Ethics Committee (HREC) under application number HREC/2018/QPCH/44293. All participants provided written informed consent to participate in the study, using the current HREC-approved study-specific Participant Information and Consent Form (PICF). When written consent could not be obtained, verbal consent was documented using the HREC-approved tele-consent form, consistent with the current PICF, and witnessed by an independent person. The study employed a combined retrospective–prospective design.

### 2.2. Peripheral Blood Mononuclear Cell Separation

Peripheral blood samples were collected after written informed consent or tele-consent was obtained. Peripheral blood mononuclear cells (PBMC) were separated by density gradient centrifugation at 500× *g* for 30 min at room temperature (RT) using Lymphoprep™ (StemCell Technologies, Tullamarine, Australia). After two washes in RPMI 1640 (Gibco, Thermo Fisher Scientific, Seventeen Mile Rocks, Australia) supplemented with 2% heat-inactivated fetal bovine serum (FBS) (Gibco) and penicillin/streptomycin/L-glutamine (R2 media), cells were counted by trypan blue exclusion, then combined with an equal volume of freezing buffer made of FBS containing 15% dimethyl sulfoxide (Sigma, Merck Life Science, Bayswater, Australia) and stored in liquid nitrogen until analysis. Cryopreservation duration varied between samples according to collection date and batch processing schedule; however, all samples were stored in liquid nitrogen until analysis and were processed using the same thawing and downstream assay workflows. For telomere length measurement, cryopreserved PBMC were thawed and processed either directly for Flow-FISH or for high-molecular-weight DNA extraction prior to Telo-Seq. Following thawing, PBMC viability was assessed by trypan blue exclusion before downstream analysis. Samples with sufficient viable cell recovery for Flow-FISH or high-molecular-weight DNA extraction were processed further. Healthy control and patient samples were processed using the same PBMC isolation, cryopreservation, thawing and assay workflows to minimise pre-analytical variation. Where possible, samples were processed in batches that included both control and patient samples, and the same protocols, reagents and instrument settings were applied across batches.

Samples from healthy individuals were supplied by the Australian Red Cross Blood Service and by hospital staff. Patient samples correspond to a small cohort of patients undergoing clinical evaluation for interstitial lung disease. The total clinical cohort comprised 24 patients, including 21 with pulmonary fibrosis and 3 with silicosis. Cross-platform analyses included 22 patients, comprising 19 with pulmonary fibrosis and 3 with silicosis. Pulmonary fibrosis–specific classification analyses included all 21 patients with pulmonary fibrosis.

Isolation of PBMC was performed on the day for hospital staff and patients, or within 24 h of the blood draw for the Red Cross and the decentralised clinical workflow samples. Healthy controls ranged in age from 18 to 78 years and were approximately evenly distributed across decade-based age groups.

### 2.3. Collection of Human Whole Lung Lavage and Cryopreservation of Alveolar Cells

Whole lung lavage (WLL) fluid was collected from consenting patients undergoing WLL at our hospital as a trial treatment for silicosis [11]. The lavage fluid was collected sequentially into 3-litre containers and returned immediately from the theatre to our on-site laboratory for processing. The fluid was centrifuged at 800× *g* for 8 min, the cell pellets pooled and resuspended in R2 media and mixed 1:1 with freezing buffer. Alveolar cells were cryopreserved in liquid nitrogen until later use.

Information on alveolar cellular composition was obtained from clinically indicated bronchoalveolar lavage (BAL) samples analysed by the hospital pathology service as part of routine clinical care. These data provided clinical context for the expected cellular composition of lavage-derived samples. The WLL samples analysed by Telo-Seq were not purified, sorted or enriched for specific cell subsets prior to DNA extraction. Therefore, WLL telomere length measurements should be interpreted as bulk measurements from mixed lavage-derived cell populations, with expected predominance of alveolar macrophages but possible contributions from lymphocytes, neutrophils, eosinophils and epithelial cells. Available BAL differential cell counts for the patients included in the WLL analysis are shown below (Table 1).

### 2.4. Isolation of Cells from Explanted Tissue

Explanted lung tissue was obtained from consented patients undergoing lung transplantation at The Prince Charles Hospital. Following surgical explantation, lungs were transported to our on-site laboratory and processed immediately, with an ischaemia time of less than 2 h. For each explanted lung, tissue was sampled systematically from the upper, middle and lower lobes. Tissue samples were mechanically minced with scissors and incubated in digestion media (0.1 g of collagenase I, dispase, and DNase I in 100 mL of RPMI 1640) for one hour at 37 °C on a MACSmix™ tube rotator (Miltenyi Biotec, Bergisch Gladbach, Germany) at maximum speed. Red blood cells were lysed by resuspending in red blood cell lysis buffer (RCLB; NH_4_Cl + NaHCO_3_ + EDTA), then washed with R2. The disaggregated cells were filtered through a 100 µm Corning^®^ cell strainer (Sigma-Aldrich, St. Louis, MO, USA), centrifuged at 500× *g* for five minutes, and resuspended in R2 media. Cells were counted by trypan blue exclusion and cryopreserved in freezing buffer for future analysis. Cell yields typically ranged from approximately 60 to 130 million cells per lobe. For Telo-Seq analysis, cells isolated from the upper lobe were used for all three patients. Tissue sections were selected as representative explant samples; however, paired histopathological quantification of fibrotic versus non-fibrotic regions and cellular composition was not performed for the tissue pieces used for Telo-Seq. Accordingly, explant-derived telomere length measurements should be interpreted as bulk tissue estimates from heterogeneous lung cell populations.

### 2.5. Telomere Measurement by Flow-FISH

Relative telomere length (RTL) was measured in duplicate by flow cytometry–fluorescence in situ hybridisation (Flow-FISH), using the peptide nucleic acid (PNA) Kit/FITC for flow cytometry (DAKO, Agilent Technologies, Santa Clara, CA, USA) as previously described by our group [12]. Cryopreserved PBMC were thawed, treated with RCLB, counted by trypan blue exclusion and washed in phosphate-buffered saline (PBS). For each assay, patient or control PBMC were combined at a 1:1 ratio with the 1301 reference cell line (Sigma-Aldrich catalogue no. 85112105), which served as an internal control for telomere fluorescence normalisation. Cell suspensions were incubated in hybridisation buffer either with or without the FITC-labelled telomere-specific PNA probe. DNA denaturation was performed at 82 °C for 10 min, followed by overnight hybridisation at RT. Cells were then washed, counterstained for DNA content and acquired on an LSRFortessa flow cytometer (BD Biosciences, Franklin Lakes, NJ, USA).

To support inter-run consistency, premixed MESF fluorescence calibration beads (Bangs Laboratories, Fishers, IN, USA) were used to assess the linearity of the FL1 fluorescence channel, and instrument settings were saved and reapplied across subsequent runs. PBMC and 1301 reference-cell populations were identified using forward- and side-scatter characteristics, followed by fluorescence-based gating on FL3-H versus FL1-H dot plots. Background fluorescence was subtracted using paired no-probe controls. RTL was calculated from the telomere fluorescence signal of sample cells relative to the 1301 control cell line, adjusted for DNA index, according to the manufacturer’s recommended formula.

RTL = [(mean FL1 sample cells with probe − mean FL1 sample cells without probe) × DNA index of control cells × 100]/[(mean FL1 control cells with probe − mean FL1 control cells without probe) × DNA index of sample cells]

### 2.6. Telomere Measurement by Telo-Seq Protocol

#### 2.6.1. DNA Sample Preparation and Nanopore Sequencing

High-molecular-weight (HMW) DNA was extracted from PBMC (4 × 10^6^ cells) using the Monarch^®^ HMW DNA Extraction Kit for Cells & Blood (NEB #T3050S/L) following the manufacturer’s instructions with minor modifications. The cell pellet was resuspended in the Nuclei Prep Solution (NEB #T3050S/L) and incubated at RT for 6 min. After addition of Nuclei Lysis Solution (NEB #T3050S/L), samples were incubated at 56 °C for 15 min in a thermal mixer set to 1500 rpm. The HMW DNA was eluted in 155 µL of elution buffer (NEB #T3050S/L) and incubated overnight at RT before further handling. The HMW DNA solution was gently pipetted up and down 10 times using a P200 with a regular pipette tip. DNA purity and concentration were assessed using a NanoDrop One spectrophotometer (Thermo Fisher Scientific) and Qubit fluorometer (Thermo Fisher Scientific), respectively. DNA fragment-size distribution was assessed using a Genomic DNA ScreenTape Kit on a 4150 TapeStation System (Agilent Technologies, Santa Clara, CA, USA).

Saliva (0.5–1 mL) was collected in a 50 mL Falcon, diluted with 2 mL of ice-cold PBS, vortexed, and centrifuged at 1000× *g* for 2 min at 4 °C to pellet the cells. The pellet was washed with 2 mL of ice-cold PBS, and the supernatant was carefully removed, leaving approximately 50 µL. HMW DNA was extracted using the same kit as above with minor modifications. Specifically, the cell pellet was resuspended in Nuclei Prep Solution (NEB #T3050S/L) and incubated at RT for 3 min, followed by incubation at 56 °C for 15 min in a thermal mixer with Nuclei Lysis Solution (NEB #T3050S/L). Lysates were centrifuged at 15,000× *g* for 3 min at RT to remove debris, after which the remaining steps were performed as described for PBMC.

Extracted DNA samples were assessed against predefined quality-control criteria before downstream use. DNA yield ranged from 7 to 10 µg per sample.

DNA purity was assessed by NanoDrop, with acceptable A_260_/A_280_ ratios of 1.8–2.0 and A_260_/A_230_ ratios > 2.0. DNA quantification was confirmed by Qubit fluorometry, with NanoDrop-to-Qubit concentration ratios generally within 1–2-fold agreement. DNA integrity was assessed using TapeStation fragment-size profiles. DNA integrity number (DIN) was not measured for every sample; therefore, DNA integrity was interpreted using available TapeStation data, with prior protocol validation supporting an expected average fragment size > 30 kb and DIN > 8.5 for PBMC-derived HMW DNA processed under these conditions. Samples that did not meet DNA quality or yield requirements were not advanced to sequencing.

For 12-plex nanopore library preparation, 5–7 µg of HMW DNA per sample was used. Nanopore sequencing libraries for Telo-Seq were prepared using the Oxford Nanopore Community Protocol for Telomere multiplex sequencing (Telo-Seq) from DNA using EXP-NBA114, EXP-ULA001, EXP-LFB001, and EXP-AUX003 (V T2T_9222_v114_revA_21May2025) with minor modifications. Telomere-adapted samples were incubated with EcoRV-HF (NEB, R3195 S/L) at 37 °C for 40 min, followed by heat inactivation at 65 °C for 20 min in a thermal cycler. Finally, adapter ligation and clean-up were performed before eluting the DNA library in 25 µL (for MinION only). The MinION loading mix consisted of Sequencing Buffer (SB—37.5 µL), Library Beads (LIB—13.5 µL) and DNA library (24 µL). For PromethION sequencing, standard procedures were followed for DNA library elution and flow-cell loading. Libraries were loaded onto either PromethION or MinION flow cells and sequenced for 72 h. Both MinION and PromethION flow cells were evaluated, and PromethION produced substantially more telomeric reads than required for telomere analysis at a 12-plex level, yielding 20–50k telomeric reads per sample, well above what is needed for reliable telomere length estimation. Therefore, the sequencing was standardised on MinION with 8–12-plex multiplexing, which consistently produced 2–5k telomeric reads per sample depending on sample type and flow cell condition at run initiation.

Flow cells were used only if they passed pre-run quality control, defined as >1200 active pores at loading, exceeding the Oxford Nanopore minimum recommendation of 800 pores. Samples were multiplexed while maintaining sufficient telomeric read depth for stable telomere length estimation. For global telomere length analysis, samples were required to generate at least 800 high-quality telomere-containing reads after wf-teloseq filtering (see below), exceeding the Oxford Nanopore-recommended minimum of 500 telomeric reads. All samples included in the final analysis generated analysable Telo-Seq data and passed sequencing quality-control thresholds, with no technical failures.

#### 2.6.2. Sequencing Data Processing and Telomere Analysis

Raw sequencing data in POD5 format were processed, and telomere analyses were performed on the high-performance computing (HPC) cluster at The University of Queensland using the Oxford Nanopore Technologies EPI2ME Labs workflow wf-teloseq (version 1.0.4) (https://github.com/epi2me-labs/wf-teloseq (accessed on 20 January 2026)). Briefly, wf-teloseq is a Nextflow-based pipeline that performs basecalling, demultiplexing of barcoded samples, and downstream telomere analysis.

Basecalling and demultiplexing were carried out using the basecalling.sh script with default parameters and the Dorado basecaller (version 1.0.1) (https://github.com/nanoporetech/dorado (accessed on 6 June 2025)). Basecalling was performed using the super-accuracy model (dna_r10.4.1_e8.2_400bps_sup@v5.2.0) with a minimum Q score threshold of 10. The resulting BAM files were converted to FASTQ format using bedtools (version 2.31.1) (bedtools bamtofastq) to facilitate downstream processing, as FASTQ is a standard sequencing file format. For samples sequenced across multiple runs, FASTQ files were concatenated prior to telomere analysis.

The telomere-analysis workflow was executed using Nextflow (version 23.04.2) with Singularity containers using the following command:

nextflow run wf-teloseq/main.nf \

--fastq <Fastq_input_sample_directory> \

--reference <directory_to_HG002qpMP_reference.fasta.gz> \

--alignment_threads 12 \

--out_dir <directory_to_output> \

-profile singularity

Sequencing depth was assessed using the number of high-quality telomere-containing reads retained after wf-teloseq quality control and filtering, rather than total raw sequencing output. This approach was used because raw read counts do not necessarily reflect the effective depth of telomeric signal available for telomere length estimation.

Although the workflow was run in reference alignment mode using a T2T HG002-derived telomeric reference, the present study focused on global telomere length estimation only. Therefore, global telomere length was estimated using the unmapped telomere-containing read output generated by wf-teloseq, representing the global telomeric read pool. The number of telomeric reads per sample was checked against the manufacturer’s recommended minimum threshold of approximately 500 reads for global telomere length estimation (Oxford Nanopore Technologies, Oxford, UK, Telo-Seq Know-How Document version 4, May 2025). In this study, samples were required to exceed a more conservative internal threshold of 800 high-quality telomere-containing reads before inclusion in downstream statistical analysis. In addition, the stability of telomere length estimates across the observed higher read-count range was empirically assessed, demonstrating consistent estimation performance (Supplementary Figure S1).

### 2.7. Telomere Length Classification

Age-adjusted telomere length centiles were derived separately for Flow-FISH and Telo-Seq using telomere length data from the healthy control cohort. Flow-FISH and Telo-Seq analyses were performed independently, and assay results were compared only after primary data processing was completed. For each assay, telomere length was modelled as a function of age, and the age-adjusted 10th percentile was used as the prespecified threshold for classifying short telomeres. Because Flow-FISH and Telo-Seq generate measurements on different scales, relative telomere length for Flow-FISH and absolute base-pair telomere length for Telo-Seq, assay-specific percentile curves were generated rather than applying a shared numerical cut-off. Patients were classified as having short telomeres when their measured telomere length fell below the assay-specific age-adjusted 10th percentile. As an exploratory sensitivity analysis, we also examined stricter age-adjusted percentile thresholds where feasible. However, because the healthy reference cohort was modest in size, particularly at the extremes of age, the 1st- and 5th-percentile estimates were considered less stable than the 10th-percentile threshold. Therefore, the 10th percentile was retained as the primary prespecified threshold for classification, while stricter cut-offs were interpreted cautiously.

### 2.8. Statistical Analysis

All statistical analyses were performed in R (version 4.5.1). Age-adjusted lower reference limits were estimated using linear quantile regression at the 10th percentile (τ= 0.10) with the quantreg package (version 6.1).

For Telo-Seq, telomere length (TL) was defined as the median read-level telomere length per sample following outlier removal using the interquartile range (IQR) method, whereby values outside 1.5 × IQR were excluded.

Flow-FISH measurements were expressed as relative telomere length (RTL). Samples were classified as healthy controls or patients based on predefined identifiers.

Age-adjusted *z*-scores were calculated separately for each assay using ordinary least squares (OLS) regression models fitted to healthy controls:

$$z=\frac{X-(\beta_{0}+\beta_{1}\times Age)}{\sigma_{residual}}$$

where X represents TL or RTL depending on the assay, $\beta_{0}\;$ and $\beta_{1}\;$ denote the regression intercept and slope, respectively, and $\sigma_{residual}\;$ is the residual standard deviation.

Model assumptions were assessed using standard diagnostic procedures, including tests for residual normality (Shapiro–Wilk, Anderson–Darling, and Lilliefors tests using the nortest package, version 1.0.4) and homoscedasticity (Breusch–Pagan test using the lmtest package, version 0.9.40).

The relationship between Telo-Seq and Flow-FISH measurements was evaluated using Pearson correlation coefficients (r) and Spearman rank correlation coefficients (ρ).

Agreement between methods was assessed using Bland–Altman analysis on the age-adjusted *z*-score scale, including estimation of the mean bias and limits of agreement (mean ± 1.96 standard deviations). Agreement was further quantified using the intraclass correlation coefficient (ICC), calculated with a two-way mixed-effects model for absolute agreement of single measurements [ICC(A,1)] using the irr package (version 0.84.1).

Samples were classified as having TL below or above the assay-specific age-adjusted 10th-percentile threshold (z <−1.28). Classification agreement between methods was assessed using Cohen’s kappa statistic with the psych package (version 2.6.5), with 95% confidence intervals derived from the asymptotic standard error.

Inter- and intra-assay variability were evaluated using the coefficient of variation (CV), defined as the standard deviation divided by the mean. Exact 95% confidence intervals for the CV were calculated using the method of Vangel, with normality of replicate measurements confirmed by Shapiro–Wilk testing.

Ninety-five per cent confidence intervals for the proportion of patients classified below the threshold were calculated using the Wilson score method, implemented in the binom package (version 1.1.1.1).

Exploratory paired tissue comparisons were performed to assess within-donor differences in telomere length between biospecimen types. For each comparison, paired differences were calculated as Tissue A minus Tissue B, such that positive values indicated longer telomeres in Tissue A. Mean paired differences were reported in base pairs with 95% confidence intervals. Paired *t*-tests were used to assess within-donor differences, and effect sizes were reported as Cohen’s d_z_ for paired samples. Given the small number of paired samples available for these analyses, particularly in the silicosis and pulmonary fibrosis cohorts, these comparisons were considered exploratory and hypothesis-generating.

Data visualisation was performed using ggplot2 (version 4.0.3), and figures were assembled using patchwork. All statistical tests were two-sided, and *p*-values < 0.05 were considered statistically significant.

## 3. Results

### 3.1. Age-Adjusted Telomere Length Distributions in an Australian Cohort

**Figure 1:**
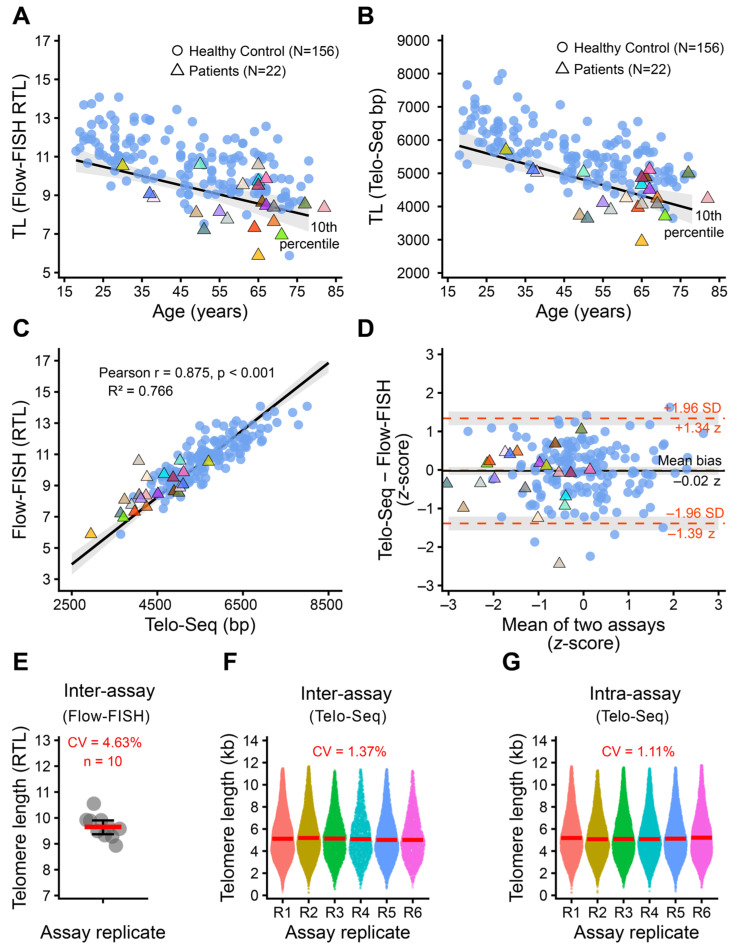
Cross-platform comparison of telomere length measurement by Flow-FISH and Telo-Seq. (**A**,**B**) Age-dependent TL decline in 156 healthy controls (blue circles) and 22 patients (coloured triangles), comprising 19 with pulmonary fibrosis and 3 with silicosis, aged 18–78 years, measured by (**A**) Flow-FISH, expressed as relative telomere length (RTL) and (**B**) Telo-Seq, expressed in base pairs (bp). Black lines indicate the 10th percentile reference cut-off estimated by linear quantile regression (τ = 0.10) fitted to healthy controls only; grey ribbons indicate the 95% confidence bands. (**C**) Correlation between paired Flow-FISH and Telo-Seq measurements in 178 participants, comprising 156 healthy controls and 22 patients. The solid black line represents the inverse linear mapping from Flow-FISH (RTL) to Telo-Seq (bp) fitted on healthy controls; the grey ribbon indicates the 95% confidence band. Pearson r = 0.875 (*p* < 0.001), and the coefficient of determination R^2^ = 0.766. (**D**) Bland–Altman analysis was performed using age-adjusted z-scores, with each assay standardised against the healthy control age-adjusted mean and residual standard deviation as described in Section 2. The solid black line indicates the mean bias (−0.02 z); the dashed red lines indicate the 95% limits of agreement (±1.96 SD: −1.39 z and +1.34 z); grey ribbons indicate the 95% confidence intervals around the mean bias and each limit of agreement. (**E**–**G**) Assay variability. (**E**) Flow-FISH inter-assay variability across *n* = 10 replicates of a single reference sample (grey circles) measured on multiple occasions (Q1–Q3 whiskers; red bar, mean). (**F**) Telo-Seq inter-assay variability across six independent sequencing runs performed on different days and flow cells. Replicate 1 (R1) was sequenced on a PromethION flow cell, whereas replicates 2–6 (R2–R6) were sequenced on MinION flow cells. (**G**) Telo-Seq intra-assay variability across six technical replicates from a single library (R1–R6). In (**F**,**G**), red horizontal bars indicate the per-replicate median. CV values were calculated from replicate median telomere lengths.

We measured leukocyte telomere length (LTL) using Flow-FISH in 156 healthy individuals aged 18–78 years to establish an age-adjusted reference range for this cohort. LTL, expressed as relative telomere length (RTL) scaled to a control cell line, showed a clear inverse relationship with age across the population (Figure 1A). Using this reference, telomere length below the age-adjusted 10th percentile was used to define short telomeres for this study, consistent with the percentile-based interpretation in clinical telomere testing. Given the moderate size of the healthy reference cohort, the 10th percentile was selected as a conservative lower-range threshold for comparative assay benchmarking rather than as a definitive diagnostic cut-off. We then applied this reference framework to a cohort of 22 patients, comprising 19 with pulmonary fibrosis and 3 with silicosis, and identified a subset of patients with LTL below the age-adjusted 10th percentile.

To enable comparison with sequencing-based measurements, we generated an independent age-adjusted reference curve for Telo-Seq, expressed in absolute base pairs, using the same healthy cohort. This allowed classification of telomere length relative to age-matched controls within each assay. High-molecular-weight genomic DNA from each participant was sequenced on the Oxford Nanopore platform, and samples were multiplexed to optimise flow-cell capacity while maintaining sufficient read depth for accurate telomere quantification. An age-adjusted 10th-percentile threshold was calculated for the Telo-Seq data to identify those with short telomeres (Figure 1B).

Residual diagnostic analyses indicated good overall model fit for both the Flow-FISH and Telo-Seq age-adjusted reference models. For Flow-FISH, residuals were centred around zero without a marked age-dependent pattern and showed no evidence of heteroscedasticity by Breusch–Pagan testing (*p* = 0.368). Residual normality was acceptable by the Shapiro–Wilk test (W = 0.985, *p* = 0.091), although the more tail-sensitive Anderson–Darling and Lilliefors tests suggested mild tail departures from normality in the distribution tails (*p* = 0.015 and *p* = 0.034, respectively; Supplementary Figure S2). For Telo-Seq, the age-adjusted model showed the expected decline in telomere length with age (slope = −30.22 bp/year, *p* = 5.1 × 10^−21^). Residuals were centred around zero, with no major residual age-dependent trend. Residual normality was acceptable by the Shapiro–Wilk, Anderson–Darling and Lilliefors tests (*p* = 0.154, *p* = 0.119 and *p* = 0.088, respectively), and there was no evidence of heteroscedasticity (Breusch–Pagan *p* = 0.275) (Supplementary Figure S3). These findings supported the use of age-adjusted models to derive assay-specific percentile thresholds.

To assess feasibility in a clinical setting, we evaluated a workflow incorporating remote blood collection, sample transport and centralised processing. In this model, patients were tele-consented, and blood samples were collected at local pathology services before shipment to our laboratory for processing and telomere length measurement.

To assess whether the decentralised clinical workflow produced DNA of sufficient quality for Telo-Seq, we compared DNA yield and purity metrics from PBMC samples collected and processed locally and from samples collected remotely and shipped to the laboratory. DNA extracted from the decentralised workflow samples (dc1 and dc2) showed A_260_/A_280_ ratios of 1.86 and 1.84, A_260_/A_230_ ratios of 2.40 and 2.37, Qubit concentrations of 157 ng/µL and 86.5 ng/µL, and total yields of 24 µg and 13 µg, respectively (Supplementary Table S1). These values were comparable to those obtained from locally processed PBMC samples and exceeded the DNA input required for Telo-Seq library preparation. All samples processed under these conditions generated analysable sequencing data with no technical failures. These results demonstrate the feasibility of implementing Telo-Seq in clinical settings where centralised sample collection is not feasible.

### 3.2. Agreement and Analytical Variability of Telo-Seq and Flow-FISH

We compared telomere length measurements obtained by Telo-Seq and Flow-FISH across the study cohort. The two assays showed a strong positive correlation (Pearson r = 0.875; R^2^ = 0.766; Figure 1C), although there was variability at the individual level, with visible dispersion around the line of best fit. Flow-FISH produced higher LTL values than Telo-Seq in the lower and mid-range of the distribution.

Agreement analysis performed on age-adjusted z-scores demonstrated minimal average bias between Telo-Seq and Flow-FISH, with a Bland–Altman mean bias of −0.02 z-score units (95% CI, −0.128 to 0.078) and 95% limits of agreement from −1.39 to 1.34 z-score units (Figure 1D and Supplementary Table S2). The intraclass correlation coefficient was 0.80 (95% CI, 0.74–0.84), indicating good agreement, while Cohen’s κ for classification below the assay-specific 10th percentile was 0.55 (95% CI, 0.36–0.74), indicating moderate categorical agreement (Supplementary Table S2). This indicates good overall agreement between the assays after age adjustment. However, the spread of differences across the range of mean z-scores shows that individual-level variation remains, particularly near lower telomere length values. These findings support the interpretation that Telo-Seq and Flow-FISH provide broadly concordant age-adjusted telomere length estimates at the group level.

Assay variability was assessed independently for each assay. When samples from a single donor were assayed by Flow-FISH over multiple days, the inter-assay coefficient of variation (CV) was 4.63% (95% CI, 3.18–8.47%; *n* = 10; Figure 1E and Supplementary Table S3). Telo-Seq demonstrated low inter-assay variability, with a CV = 1.37% (95% CI, 0.86–3.36%; *n* = 6) across six independent sequencing runs performed on different days using PromethION for replicate 1 (R1) and MinION for replicates 2–6 (R2–R6) (Figure 1F and Supplementary Table S3). Intra-assay variability was assessed by sequencing replicate libraries from the same DNA preparation on a single MinION flow cell, resulting in a CV of 1.11% (95% CI, 0.69–2.72%; *n* = 6; Figure 1G and Supplementary Table S3).

### 3.3. Discordant Classification of Short Telomeres Between Assays

We compared telomere length classification in 21 patients with pulmonary fibrosis who had paired Flow-FISH and Telo-Seq measurements. The cohort was predominantly male (85.7%) and had a mean age of 64.6 years, consistent with the typical demographic profile of PF. Using assay-specific, age-adjusted 10th-percentile thresholds, Flow-FISH classified 9 of 21 patients as having short telomeres (42.9%; 95% CI, 24.5–63.5%), compared to 11 of 21 patients as having short telomeres by Telo-Seq (52.4%; 95% CI, 32.4–71.7%).

**Figure 2:**
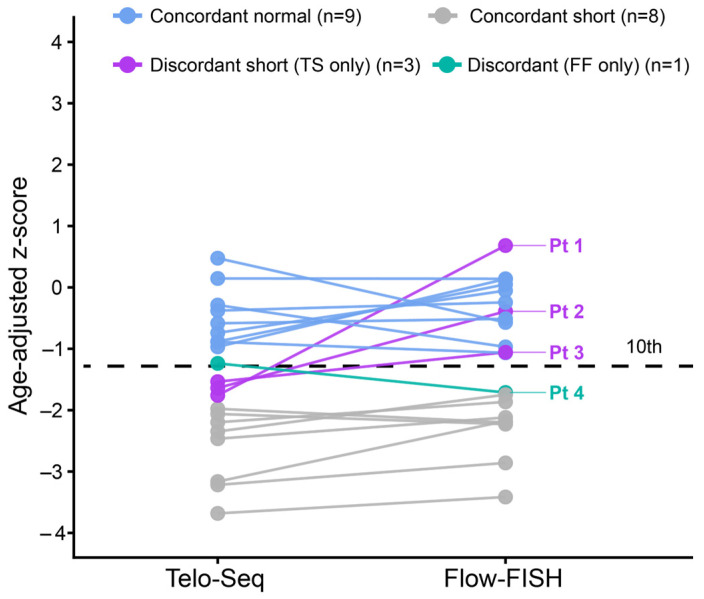
Age-adjusted classification concordance between Flow-FISH and Telo-Seq in patients with pulmonary fibrosis. Paired age-adjusted z-scores for Telo-Seq and Flow-FISH are shown for each patient with pulmonary fibrosis. The dashed line indicates the 10th-percentile threshold. Short-telomere classification was defined relative to assay-specific age-adjusted 10th-percentile thresholds. Concordant and discordant classifications are highlighted, including three patients classified as short by Telo-Seq only and one patient classified as short by Flow-FISH only.

Categorical classification was concordant (above or below the 10th percentile in both assays) in 17 of 21 patients. Eight patients were classified as short by both assays, while nine were classified as within the normal range by both assays. Four patients showed discordant classification. Three were classified as short by Telo-Seq but within the normal range by Flow-FISH, whereas one was classified as short by Flow-FISH but within the normal range by Telo-Seq (Figure 2). In the latter case, the Telo-Seq value was close to the assay-specific age-adjusted 10th-percentile threshold, suggesting the discordant classification may reflect assay variability around the cut-off. Telomere length values and percentile-based classifications for each discordant case are shown in Table 2. Flow-FISH values are reported in relative telomere length units, whereas Telo-Seq values are reported in base pairs; classification was determined using assay-specific, age-adjusted 10th-percentile thresholds.

Clinical genetic testing was performed where indicated by a commercial clinical testing laboratory using a validated interstitial lung disease gene panel. Within the assay-discordant subgroup, reportable variants were identified in two of four patients: an *RTEL1* variant in Pt1 and an *SMPD1* variant in Pt2. The *RTEL1* variant provides relevant telomere-biology context [13], whereas the *SMPD1* variant is included as clinical genetic context only. No reportable variants were identified in Pt3 or Pt4. Clinical and genetic information for the four discordant cases is provided in Table 3.

### 3.4. Telomere Length Measurement Across Multiple Tissue Types

**Figure 3:**
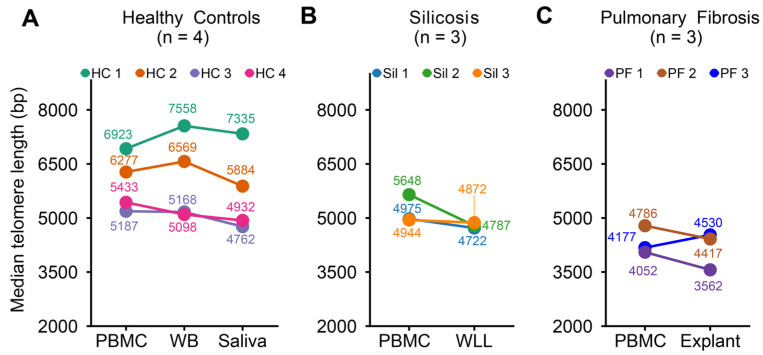
Telo-Seq enables telomere length measurement across multiple human tissue sources. Paired median telomere length measurements are shown for (**A**) healthy controls across PBMC, whole blood (WB) and saliva, (**B**) patients with silicosis across PBMC and whole lung lavage (WLL) and (**C**) patients with pulmonary fibrosis across PBMC and explanted lung tissue. Lines connect biospecimens from the same individual. Collection intervals between tissue samples are detailed in Table 4. Exploratory paired tissue comparisons are provided in Supplementary Table S4. These tissue-based analyses are exploratory and should be interpreted cautiously given the small sample size, variable pairing intervals and mixed cellular composition of lung-derived samples.

Telo-Seq can quantify telomere length across multiple tissue types, provided sufficient high-molecular-weight DNA can be extracted, although prior studies have been largely limited to PBMC, fibroblasts, and cancer samples [10,14]. To explore this capability in the context of lung pathology, we applied Telo-Seq to multiple biospecimens from our human biobank, including PBMC, whole blood, saliva, whole lung lavage (WLL) fluid and explanted lung tissue (Figure 3 and Table 4).

Telo-Seq was successfully applied to all biospecimen types, with each sample generating sufficient telomeric reads for global telomere length estimation. In healthy controls, telomere length was broadly comparable between PBMC and whole blood, while saliva showed more variability and was generally similar to, or slightly shorter than, blood-derived measurements.

In patients with silicosis, WLL-derived samples showed shorter telomere length than paired PBMC in all three cases. In patients with pulmonary fibrosis, explanted lung tissue showed a more variable pattern, with shorter telomeres than paired PBMC in two of three cases and longer telomeres in one case. These lung-derived samples were analysed as bulk cell populations: WLL samples were macrophage-rich but not sorted or purified, and explant samples represented heterogeneous tissue-derived cell suspensions.

Exploratory paired comparisons did not show statistically significant differences between tissue types, although interpretation is limited by the small number of paired samples, variable collection intervals, mixed cellular composition and potential pre-analytical effects (Supplementary Table S4). Together, these findings demonstrate the technical feasibility of applying Telo-Seq to diverse human biospecimens, including lung-derived samples, but should be interpreted as exploratory rather than evidence of definitive tissue-specific telomere shortening.

## 4. Discussion

In this study, we evaluated a long-read sequencing-based assay for telomere length measurement in a clinical cohort and compared it directly with Flow-FISH, the current clinical reference method. Telo-Seq showed strong correlation and good overall agreement with Flow-FISH, lower assay variability and discordant classification in a small number of patients near assay-specific short-telomere thresholds. These findings suggest that sequencing-based approaches may provide a reproducible complementary method for telomere length assessment and may offer additional information on short-telomere burden compared with population-averaged fluorescence-based assays.

Accurate identification of short telomeres has increasing clinical importance in pulmonary fibrosis and related telomere biology disorders, where it may inform diagnosis, prognosis, and treatment decisions. Telomere length can be measured using several complementary approaches, each with distinct strengths and limitations (reviewed in ref. [15]). Quantitative PCR is inexpensive, scalable and requires low DNA input, but provides only a relative average telomere signal and is sensitive to pre-analytical and inter-laboratory variability. Terminal restriction fragment analysis by Southern blot provides an absolute telomere length distribution and has historically served as a DNA-based reference method, but it is labour-intensive, includes subtelomeric DNA in the measured fragment and is poorly suited to scalable clinical implementation. Higher-resolution methods such as single telomere length analysis (STELA) and telomere shortest length assay (TeSLA) can detect very short telomeres, which are biologically important because the shortest telomeres may drive DNA-damage responses and replicative senescence; however, STELA is restricted to selected chromosome ends, while TeSLA is lower throughput and does not capture the longest telomeres.

Flow-FISH remains the current gold-standard assay used clinically to support the diagnosis of short telomeres [16]; however, its analytical variability and reliance on peripheral blood measurements may limit its sensitivity in some contexts. At the lower end of the telomere length distribution, Flow-FISH measurements tended to be higher than corresponding Telo-Seq measurements, consistent with previous reports [9]. Furthermore, Flow-FISH provides a relative, population-averaged fluorescence measurement and has limited sensitivity to telomere heterogeneity, such that a subset of critically short telomeres may be masked within the average signal. In this context, long-read nanopore sequencing approaches such as Telo-Seq offer a distinct balance of features by providing absolute telomere length estimates together with chromosome arm-specific and potentially allele-specific information [10]. These differences reinforce the view that Telo-Seq is best positioned as a complementary high-resolution assay rather than a direct replacement for established clinical methods.

Classification differed between Flow-FISH and Telo-Seq in four patients with pulmonary fibrosis. Three were below the assay-specific age-adjusted 10th percentile by Telo-Seq but above the corresponding Flow-FISH threshold, while one showed the reverse pattern. This indicates that, despite good cohort-level agreement, individual classification can differ near assay-specific decision boundaries. In the patient classified as short by Flow-FISH but not by Telo-Seq, the Telo-Seq value was close to the assay-specific 10th percentile, suggesting threshold-adjacent classification rather than marked separation between methods.

These cases are presented as observed differences in assay classification, not as evidence that either method was definitively correct. Orthogonal approaches, such as terminal restriction fragment analysis by Southern blot, may help contextualise such cases in future studies, although TRF analysis also has limitations, including the measurement of subtelomeric sequence in addition to telomeric repeats.

Clinical genetic testing was performed where indicated using a validated interstitial lung disease gene panel. Among the four patients with differing classifications, Pt1 carried an *RTEL1* variant, providing relevant telomere-biology context given the established role of *RTEL1* in telomere maintenance and genome stability. Pt2 carried an *SMPD1* variant, which is included as clinical genetic context but is not interpreted as evidence of a canonical telomere-maintenance disorder. No reportable variants were identified in Pt3 or Pt4. These assay-discordant cases may have clinical implications, as assay-discordant telomere classification in suspected telomere biology disorders could affect diagnostic evaluation, family screening, transplant risk assessment and treatment planning. However, the number of discordant cases in this study was small and further work is required to determine whether these differences translate into improved clinical outcomes.

Several factors may contribute to discordant classification between the assays, including biological variability, assay-specific bias, and differences in the telomere features captured by each method. Flow-FISH reports a population-averaged fluorescence signal and may be affected by sample handling, cell viability, background fluorescence, probe hybridisation efficiency, instrument calibration, gating strategy and the contribution of longer telomeres within the same cell population to the mean signal. Conversely, sequencing-based approaches may be influenced by DNA extraction, library preparation, adapter ligation, enrichment or sequencing bias, including possible under-representation of very short telomeric molecules. These findings support cautious interpretation of threshold-based classification, particularly close to assay-specific cut-offs.

The present data do not support routine re-testing of all patients with normal Flow-FISH results. However, Telo-Seq may have value as a complementary assay in selected cases where clinical suspicion remains high despite Flow-FISH results above the assay-specific threshold. Such scenarios may include pulmonary fibrosis with early disease onset, familial disease, extrapulmonary manifestations such as cytopenia or liver disease, suggestive telomere-gene variants, or pre-transplant assessment. Nevertheless, formal clinical recommendations regarding re-testing of Flow-FISH–normal patients will require larger prospective studies with independent clinical adjudication, orthogonal validation and linkage to clinically meaningful outcomes.

A key advantage of Telo-Seq is its applicability across multiple tissue types. To our knowledge, this study represents the first application of Telo-Seq to paired human tissue samples, enabling direct comparison of telomere length between peripheral blood mononuclear cells and disease-relevant lung compartments, including whole lung lavage fluid and explanted lung tissue from the same individuals. In patients with PF, telomere length measured in explanted lung tissue was shorter than that observed in paired PBMC in two of three cases, while in patients with silicosis, telomere length measured in macrophage-rich WLL samples was consistently shorter than in paired PBMC.

These findings are biologically plausible but should be interpreted cautiously given the small sample size and the mixed cellular composition of WLL and explanted lung tissue. In PF, repeated cycles of injury and aberrant repair place a high proliferative burden on alveolar epithelial cells and fibroblast populations, leading to localised telomere shortening that may not be fully reflected in circulating leukocytes. Previous work utilising FISH staining has shown that alveolar type II cells in fibrotic regions have significantly shorter telomeres than in non-fibrotic regions, suggesting that critically short telomeres in lung cells are associated with the fibrotic process and adverse clinical outcomes [17]. Similarly, shorter telomeres in macrophage-rich WLL samples may reflect differences in cellular composition, chronic inflammation, macrophage activation and local proliferation within the injured lung microenvironment. This interpretation is consistent with studies demonstrating a proliferating macrophage population in fibrotic lungs, including in response to SARS-CoV-2 infection, adopting transcriptional programs shared with idiopathic pulmonary fibrosis [18]. The fibrotic lung is also characterised by oxidative stress, mitochondrial dysfunction, inflammation and altered extracellular matrix signalling, all of which are features of accelerated tissue ageing that may amplify DNA damage responses and contribute to accelerated telomere attrition in local tissue compartments (reviewed in ref. [19]).

Nevertheless, tissue-derived telomere length differences cannot be attributed solely to disease-related telomere shortening. Shorter telomeres in lung-derived samples may also reflect normal differences in telomere attrition rates between blood and lung tissue, independent of disease status. Because healthy lung tissue was not available for comparison, we cannot determine whether the observed differences reflect disease-specific pathology, normal tissue-specific telomere attrition, differences in cell composition, local inflammatory burden, proliferative history or pre-analytical factors. In addition, bulk Telo-Seq of WLL and explanted lung tissue cannot distinguish whether shorter telomeres arise from epithelial cells, macrophages, fibroblasts, other lung-resident populations or differences in cell-type composition. Nor can these bulk measurements fully account for differences in fibrotic burden, inflammatory state, smoking history, medication exposure or local proliferative history. Pre-analytical variables, including ischaemia time, enzymatic digestion and DNA quality, may also influence tissue-derived telomere length estimates. These observations should therefore be considered exploratory and hypothesis-generating, requiring validation in larger paired cohorts using cell-type-resolved or spatial telomere analyses.

Practical implementation of telomere testing also requires consideration of cost, workload, turnaround time and sample logistics. Flow-FISH is an established clinical assay that can be performed on standard flow-cytometry platforms, with published protocols describing the analysis of nucleated blood cells from 22 individuals over approximately 12 h distributed across 2–3 days [20]. However, clinical Flow-FISH testing is logistically constrained by the requirement for viable fresh blood or PBMC, room-temperature transport, prior booking, sample arrival within 24–36 h of collection [21] and centralised reference-laboratory processing. International clinical services also report logistical constraints, including restricted sample stability and turnaround times of several weeks [22]. In contrast, Telo-Seq requires extraction of high-molecular-weight DNA, library preparation, nanopore sequencing and bioinformatic analysis, which introduce additional technical and computational workload. A practical consideration for Telo-Seq adoption is the choice of sequencing platform and multiplexing level. In this study, both MinION and PromethION flow cells generated analysable telomere length data. PromethION produced substantially higher telomeric read counts than required for 12-plex analysis, whereas MinION with 8–12-plex multiplexing provided sufficient telomeric read depth for routine global telomere length estimation at lower per-run scale. Flow cell performance was a key determinant of sequencing success, and runs initiating with low active pore counts were more likely to generate insufficient telomeric reads.

In our laboratory, local per-sample cost estimates at the time of manuscript preparation were approximately AUD 160 for Flow-FISH and AUD 270 for Telo-Seq, including consumables and hands-on labour but excluding equipment depreciation, institutional overheads, bioinformatic infrastructure and clinical reporting. Flow-FISH was completed over approximately 2–3 days, whereas the current Telo-Seq workflow, including DNA extraction, library preparation, sequencing and bioinformatic analysis, was completed over approximately 7 days. Although Telo-Seq is therefore currently more expensive and technically more complex in our workflow, its DNA-based format enables sample batching, barcoding and multiplexed sequencing, which may improve scalability and reduce per-sample costs with further optimisation.

Therefore, although long-read telomere sequencing provides additional resolution, including absolute telomere length and chromosome arm-specific information, its clinical adoption will require further standardisation of DNA input thresholds, sequencing-depth requirements, bioinformatics pipelines, turnaround time and cost-effectiveness relative to established Flow-FISH testing.

This study has several limitations. First, although the healthy control cohort enabled construction of assay-specific age-adjusted reference curves, the clinical cohort was modest in size, particularly for pulmonary fibrosis and silicosis subgroup analyses. As a result, estimates of short-telomere classification and assay concordance and discordant classifications should be interpreted cautiously. Second, the study was cross-sectional and did not include longitudinal clinical outcomes, such as disease progression, transplant outcomes, survival, treatment tolerance or extrapulmonary complications. Therefore, the prognostic significance of Telo-Seq-derived telomere measurements could not be assessed in the present study. This includes both the global telomere length metrics reported here and additional chromosome-specific or distributional metrics that are technically enabled by the workflow but reserved for future analyses. Third, no independent diagnostic gold standard was available to adjudicate discordant cases between Flow-FISH and Telo-Seq. Although clinical and genetic findings provided useful context, the study cannot determine whether the discordance reflects erroneous classification by either assay, biological variability or assay-specific differences in what is being measured. Fourth, the findings were generated at a single centre and were not validated in an external cohort or across multiple laboratories, which will be required before broader clinical implementation.

The tissue-based analyses were exploratory and involved a small number of paired samples. Although these analyses demonstrate the feasibility of applying Telo-Seq to lung-derived biospecimens, interpretation is limited by mixed cellular composition, variable pairing intervals, and potential pre-analytical effects. In addition, although all samples generated analysable Telo-Seq data, telomeric read depth varied across biospecimens, and the impact of sequencing-depth variability on future chromosome-specific or distributional telomere metrics will require further evaluation. These factors will need to be addressed in future validation studies using larger paired cohorts and cell-type-resolved approaches.

Future studies should focus on multicentre validation, assay harmonisation and clinical outcome integration. Larger studies across independent laboratories will be required to define inter-laboratory reproducibility and establish standardised protocols for sample handling, DNA extraction, library preparation, sequencing depth, quality-control thresholds and bioinformatic analysis. Although the 10th percentile was appropriate for comparative assay benchmarking in this study, clinical interpretation of severe short-telomere syndromes may require stricter thresholds, such as the 1st or 5th percentile. Therefore, larger population reference datasets are needed to generate robust age-adjusted and potentially sex- and ancestry-adjusted Telo-Seq reference curves. Beyond median telomere length, future analyses should evaluate whether sequencing-derived features such as chromosome arm-specific telomere length, shortest-quartile telomere length and the fraction of telomeres below critically short thresholds provide additional biological or clinical information. Linking these metrics to longitudinal outcomes, including pulmonary function decline, transplant complications, treatment tolerance, extrapulmonary disease and survival, will be essential to define the clinical utility of Telo-Seq in pulmonary fibrosis and suspected telomere biology disorders. The current workflow is compatible with these analyses, and future work will explore chromosome-specific telomere lengths and the proportion of telomeres below defined critical-length thresholds in this Australian cohort.

Overall, long-read sequencing provides a reproducible and versatile approach to telomere length measurement. Its ability to identify assay-discordant short-telomere classifications and to assess telomere length in disease-relevant tissues supports further evaluation of its clinical utility. Following additional validation in larger, multicentre cohorts, sequencing-based approaches may emerge as clinically valuable complementary tools for telomere length assessment, enabling the development of assay-specific reference ranges and integration with longitudinal clinical outcomes to define their role in the management of pulmonary fibrosis and telomere biology disorders.

## 5. Conclusions

This study demonstrates the feasibility of applying long-read sequencing to telomere length assessment in a clinical setting and across multiple human tissues. By enabling direct measurement of telomeric DNA, Telo-Seq offers a complementary perspective to fluorescence-based approaches that rely on population-averaged signals.

Rather than replacing existing assays, sequencing-based methods may, following larger multicentre validation studies, complement current diagnostic frameworks by capturing telomere features that are not readily accessible using conventional techniques, including tissue-specific variation and the presence of critically short telomeres. These capabilities may be particularly relevant in fibrotic lung diseases, where telomere dysfunction is implicated in disease pathogenesis but may not be fully reflected in peripheral blood measurements.

Integration of sequencing-based telomere analysis into clinical and research workflows will require further standardisation, including assay-specific reference ranges, harmonised quality-control thresholds, and validation across tissue types. Future studies should link high-resolution telomere measurements with longitudinal clinical outcomes to determine whether these approaches improve patient stratification and therapeutic decision-making.

## Figures and Tables

**Table 1:** Clinical BAL differential cell counts available for patients included in the WLL Telo-Seq analysis.

Sample ID	% Macrophages	% Lymphocytes	% Neutrophils	% Eosinophils
Silicosis 1	66	27	7	0
Silicosis 2	64	26	9	1
Silicosis 3	69	6	18	7

**Table 2:** Telomere length classification of discordant cases.

ID	TL-Flow-FISH (RTL Units)	10th Percentile for Age	Flow-FISH z-Score	Flow-FISH Classification (Age-Adjusted 10th Percentile)	TL-Telo-Seq (bp)	10th Percentile for Age	Telo-Seq z-Score	Telo-Seq Classification (Age-Adjusted 10th Percentile)
Pt1	10.57	8.47	0.68	Above	4066	4263	−1.76	Below
Pt2	9.54	8.76	−0.39	Above	4257	4454	−1.64	Below
Pt3	8.38	8.38	−1.06	Above	4075	4199	−1.54	Below
Pt4	7.63	8.38	−1.71	Below	4248	4199	−1.24	Above

Abbreviations: RTL, relative telomere length. Flow-FISH values are reported in RTL units and should not be interpreted as percentile values. Telo-Seq values are reported in base pairs. Classification was determined relative to assay-specific age-adjusted 10th-percentile thresholds. The corresponding age-adjusted z-score threshold for the 10th percentile is −1.28.

**Table 3:** Clinical and genetic characteristics of patients with discordant Flow-FISH and Telo-Seq classification.

ID	Age	Sex	Diagnosis	Extrapulm. Features	Treatment	Gene	Variant	Zygosity	Consequence	ACMG Classification	Population Frequency, gnomAD (AC/AN)
Pt1	67	M	IPF (FH-PF)	Nil	NIN	*RTEL1*	c.524A>G, p. (Lys175Arg)	HET	Missense variant	VUS	0/0
Pt2	61	M	IPF (FH-PF)	Early greying	PIR	*SMPD1*	c.757G>C, p. (Asp253His)	HET	Missense variant	Likely pathogenic	4/249222
Pt3	69	M	IPF (FH-PF)	Early greying	NIN	ND	ND	-	-	-	-
Pt4	69	M	IPF (FH-PF)	Thrombocytopenia, early greying	NIN	ND	ND	-	-	-	-

Abbreviations: M, male; IPF, idiopathic pulmonary fibrosis; FH-PF, family history of pulmonary fibrosis; Extrapulm, extrapulmonary; NIN, nintedanib; PIR, pirfenidone; ND, not detected; HET, heterozygous; ACMG, American College of Medical Genetics and Genomics; VUS, variant of uncertain significance; gnomAD, Genome Aggregation Database; AC, allele count; AN, allele number.

**Table 4:** Demographics and Telo-Seq analysis on biospecimens from the human biobank.

ID	Age	Sex	Condition	Sample	Telomere Length (bp)	Telomeric Reads	Age-Adjusted z-Score	Interval from PBMC
				PBMC	6923	33,701	2.06	Reference
Healthy 1	44	Male	Healthy	Whole blood	7558	18,061	3.16	2 weeks after
				Saliva	7335	6677	2.77	9 months after
				PBMC	6277	3211	−0.19	Reference
Healthy 2	22	Female	Healthy	Whole blood	6569	2662	0.31	14 months after
				Saliva	5884	6277	−0.87	14 months after
				PBMC	5187	7028	−0.35	Reference
Healthy 3	55	Male	Healthy	Whole blood	5168	4870	−0.38	5 months after
				Saliva	4762	4674	−1.08	5 months after
				PBMC	5433	4219	0.23	Reference
Healthy 4	58	Female	Healthy	Whole blood	5098	6725	−0.35	14.5 months after
				Saliva	4932	8440	−0.63	14.5 months after
Silicosis 1	41	Male	Silicosis	PBMC	4975	1241	−1.44	Reference
				WLL *	4722	9120	−1.88	Within 2 years
Silicosis 2	30	Male	Silicosis	PBMC	5648	51,968	−0.86	Reference
				WLL *	4787	7578	−2.34	Within 1 year
Silicosis 3	38	Male	Silicosis	PBMC	4944	21,178	−1.65	Reference
				WLL *	4872	13,032	−1.78	Within 1 year
PF 1	55	Male	PF	PBMC	4052	2113	−2.3	Reference
				Lung explant	3562	1227	−3.15	Within 1 year
PF 2	65	Male	PF	PBMC	4786	4220	−0.52	Reference
				Lung explant	4417	1205	−1.16	On the same day
PF 3	69	Male	PF	PBMC	4177	16,916	−1.36	Reference
				Lung explant	4530	4284	−0.75	Within 2 years

* WLL, whole lung lavage. Per-sample telomere length (TL) across tissue sources, with age-adjusted TL z-scores relative to a healthy PBMC reference panel (*n* = 156). Z-scores were calculated using a regression model of TL on age (predicted TL = 7052.94 − 30.2 × Age [bp]; residual SD = 581.5 bp) and represent deviation from the expected TL at a given age. This standardisation enables comparison across individuals and tissue types with differing TL ranges. The reference was derived from healthy controls; therefore, z-scores represent deviations from the healthy PBMC reference and are not intended for clinical classification of non-PBMC tissues. Paired samples were defined as biospecimens obtained from the same individual. Collection intervals are shown relative to the paired PBMC sample from the same donor. PBMC were used as the reference sample for within-donor comparisons. Samples were not collected contemporaneously in all cases; therefore, cross-tissue comparisons should be interpreted cautiously.

## Data Availability

Processed data sufficient to reproduce the findings of this study are available at https://github.com/SRaiTPJ/Teloseq_Australian_Cohort (accessed on 20 January 2026). Due to the sensitive nature of human genomic data and ongoing analyses, raw sequencing data are not publicly available but may be shared upon reasonable request and appropriate approvals. Raw data underlying additional analyses will be made available in future publications.

## Supplementary Materials

The following supporting information can be downloaded at: https://www.mdpi.com/article/10.3390/cells15131165/s1, Supplementary Figure S1: Relationship between telomeric read count and median telomere length by Telo-Seq. Supplementary Figure S2: Residual diagnostics for the Flow-FISH age-adjusted telomere length (TL) model. Supplementary Figure S3: Residual diagnostics for the Telo-Seq age-adjusted telomere length (TL) model. Supplementary Table S1: High-molecular-weight DNA yield, purity and sequencing-read metrics from PBMC samples used for Telo-Seq, including decentralised workflow samples. Supplementary Table S2: Quantitative agreement between Telo-Seq and Flow-FISH in paired healthy control and patient samples (*n* = 178). Supplementary Table S3: Inter- and intra-assay variability of telomere length measurements. Supplementary Table S4: Exploratory paired tissue comparisons of median telomere length.

## Abbreviations

The following abbreviations are used in this manuscript:

PF	Pulmonary fibrosis
PBMC	Peripheral blood mononuclear cells
Flow-FISH	Flow fluorescence in situ hybridisation
WLL	Whole lung lavage
LTL	Leukocyte telomere length
CV	Coefficient of variation
PBS	Phosphate-buffered saline
