# Supplementary material for: Clinical Evaluation of Long-Read Sequencing for Telomere Length Assessment in Human Blood and Lung Tissues
Source: Cells. 2026 Jun 26;15(13):1165. doi: 10.3390/cells15131165 (PMC13359500; doi:10.3390/cells15131165)
Supplement: Supplementary file 1 [file cells-15-01165-s001.zip › Supplementary data .pptx]

## Slide 1
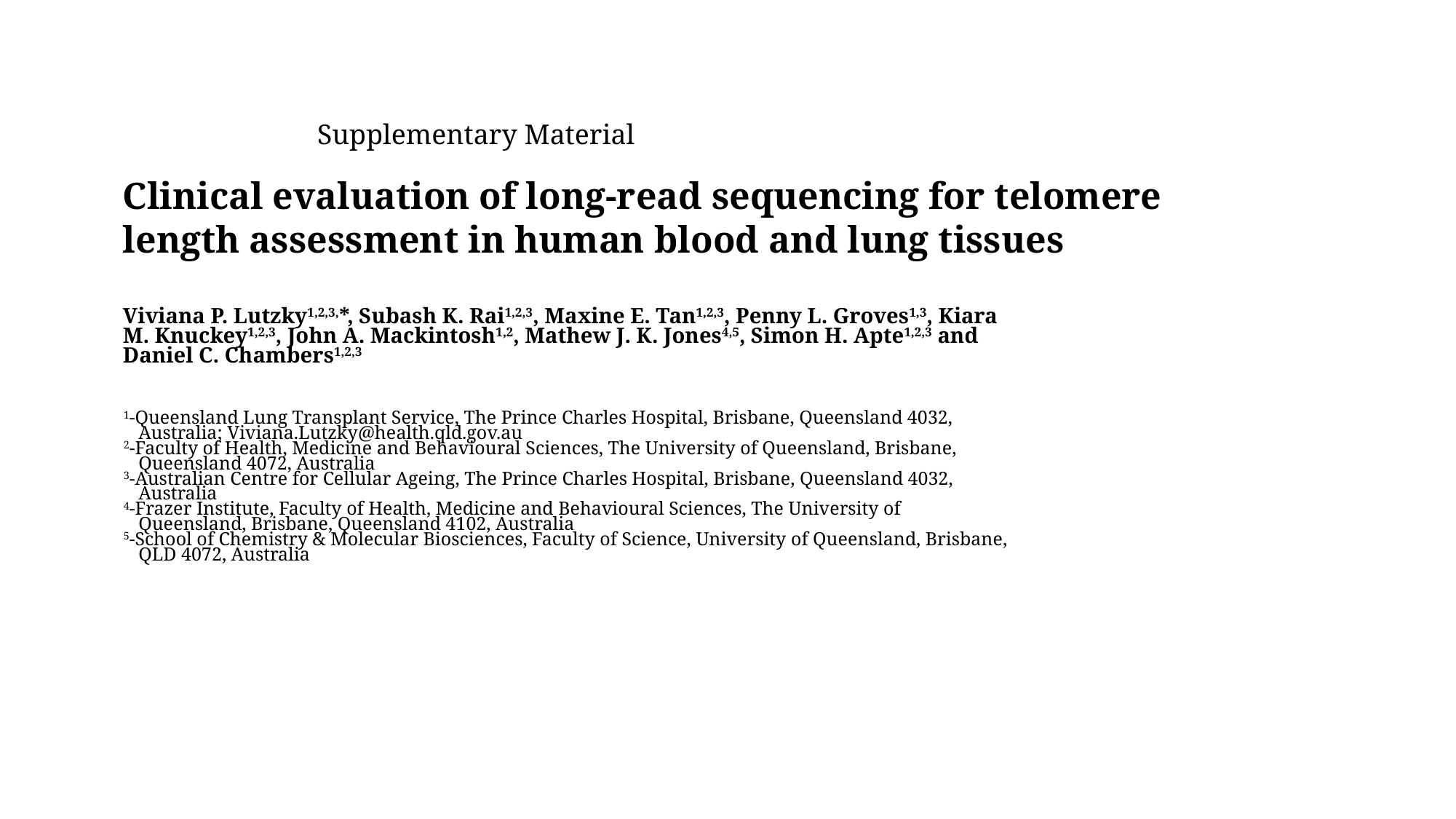

Supplementary Material
Clinical evaluation of long-read sequencing for telomere length assessment in human blood and lung tissues
Viviana P. Lutzky1,2,3,*, Subash K. Rai1,2,3, Maxine E. Tan1,2,3, Penny L. Groves1,3, Kiara M. Knuckey1,2,3, John A. Mackintosh1,2, Mathew J. K. Jones4,5, Simon H. Apte1,2,3 and Daniel C. Chambers1,2,3
1-Queensland Lung Transplant Service, The Prince Charles Hospital, Brisbane, Queensland 4032, Australia; Viviana.Lutzky@health.qld.gov.au
2-Faculty of Health, Medicine and Behavioural Sciences, The University of Queensland, Brisbane, Queensland 4072, Australia
3-Australian Centre for Cellular Ageing, The Prince Charles Hospital, Brisbane, Queensland 4032, Australia
4-Frazer Institute, Faculty of Health, Medicine and Behavioural Sciences, The University of Queensland, Brisbane, Queensland 4102, Australia
5-School of Chemistry & Molecular Biosciences, Faculty of Science, University of Queensland, Brisbane, QLD 4072, Australia

## Slide 2
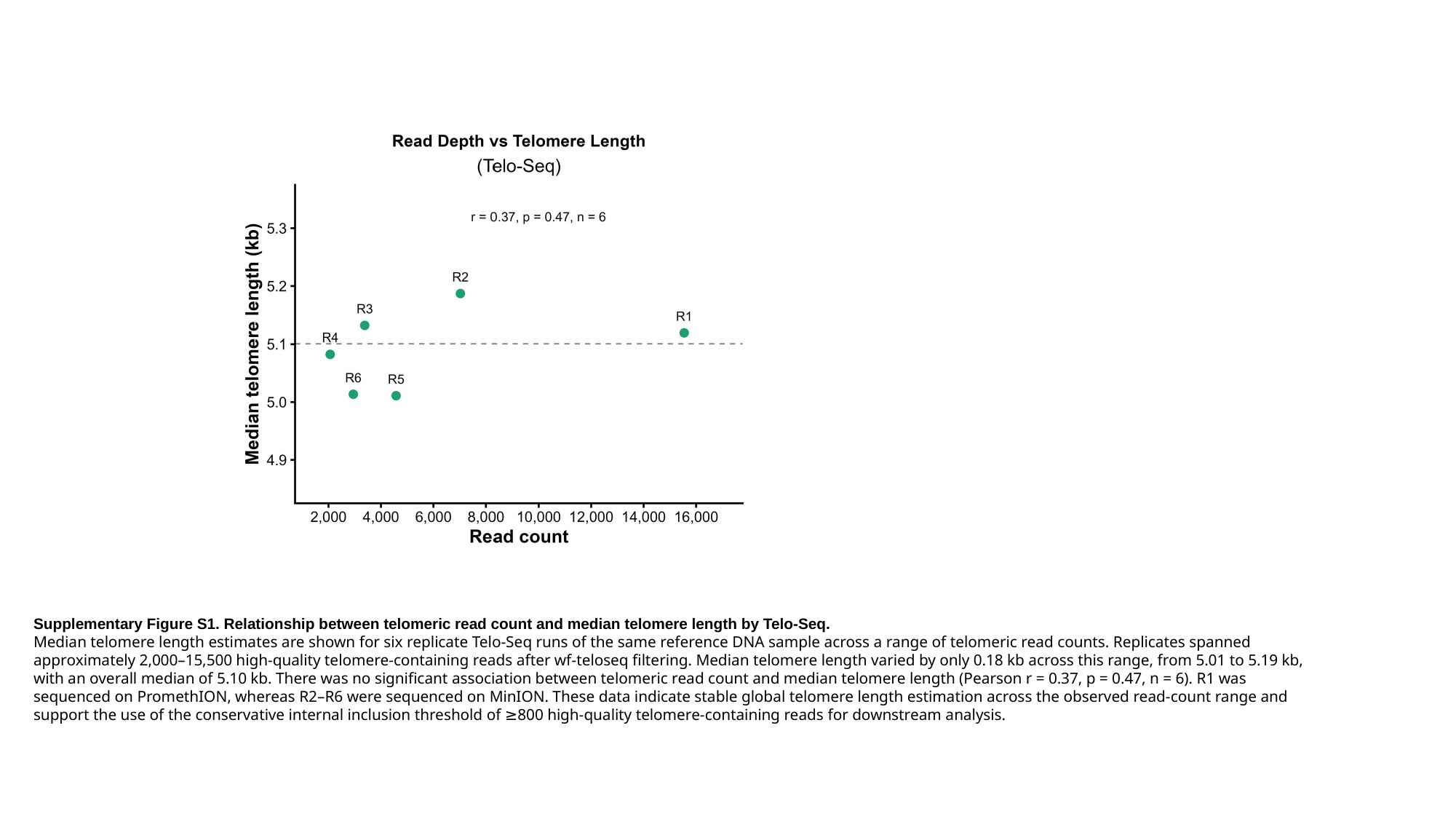

Supplementary Figure S1. Relationship between telomeric read count and median telomere length by Telo-Seq.
Median telomere length estimates are shown for six replicate Telo-Seq runs of the same reference DNA sample across a range of telomeric read counts. Replicates spanned approximately 2,000–15,500 high-quality telomere-containing reads after wf-teloseq filtering. Median telomere length varied by only 0.18 kb across this range, from 5.01 to 5.19 kb, with an overall median of 5.10 kb. There was no significant association between telomeric read count and median telomere length (Pearson r = 0.37, p = 0.47, n = 6). R1 was sequenced on PromethION, whereas R2–R6 were sequenced on MinION. These data indicate stable global telomere length estimation across the observed read-count range and support the use of the conservative internal inclusion threshold of ≥800 high-quality telomere-containing reads for downstream analysis.

## Slide 3
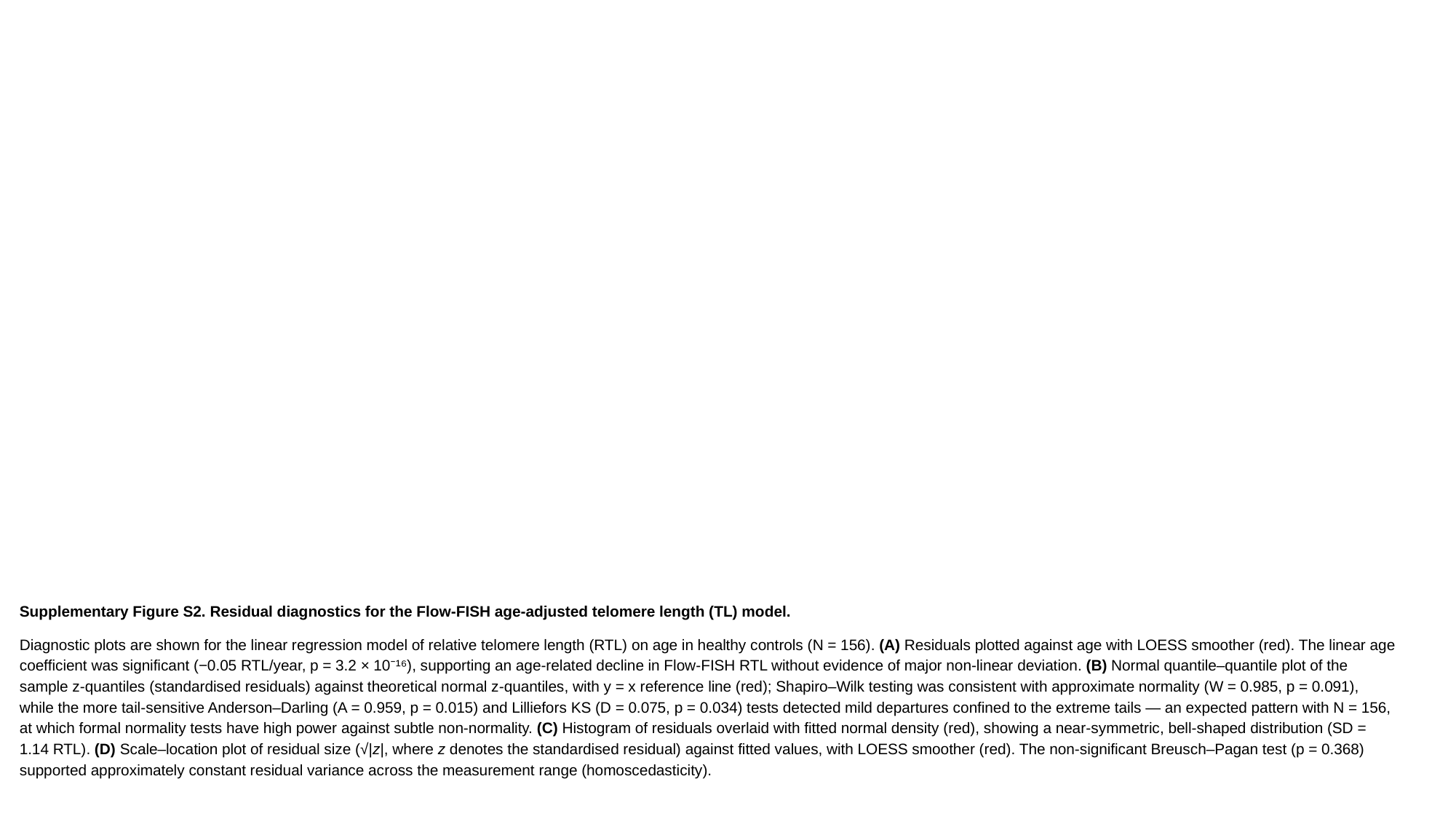

Supplementary Figure S2. Residual diagnostics for the Flow-FISH age-adjusted telomere length (TL) model.
Diagnostic plots are shown for the linear regression model of relative telomere length (RTL) on age in healthy controls (N = 156). (A) Residuals plotted against age with LOESS smoother (red). The linear age coefficient was significant (−0.05 RTL/year, p = 3.2 × 10⁻¹⁶), supporting an age-related decline in Flow-FISH RTL without evidence of major non-linear deviation. (B) Normal quantile–quantile plot of the sample z-quantiles (standardised residuals) against theoretical normal z-quantiles, with y = x reference line (red); Shapiro–Wilk testing was consistent with approximate normality (W = 0.985, p = 0.091), while the more tail-sensitive Anderson–Darling (A = 0.959, p = 0.015) and Lilliefors KS (D = 0.075, p = 0.034) tests detected mild departures confined to the extreme tails — an expected pattern with N = 156, at which formal normality tests have high power against subtle non-normality. (C) Histogram of residuals overlaid with fitted normal density (red), showing a near-symmetric, bell-shaped distribution (SD = 1.14 RTL). (D) Scale–location plot of residual size (√|z|, where z denotes the standardised residual) against fitted values, with LOESS smoother (red). The non-significant Breusch–Pagan test (p = 0.368) supported approximately constant residual variance across the measurement range (homoscedasticity).

## Slide 4
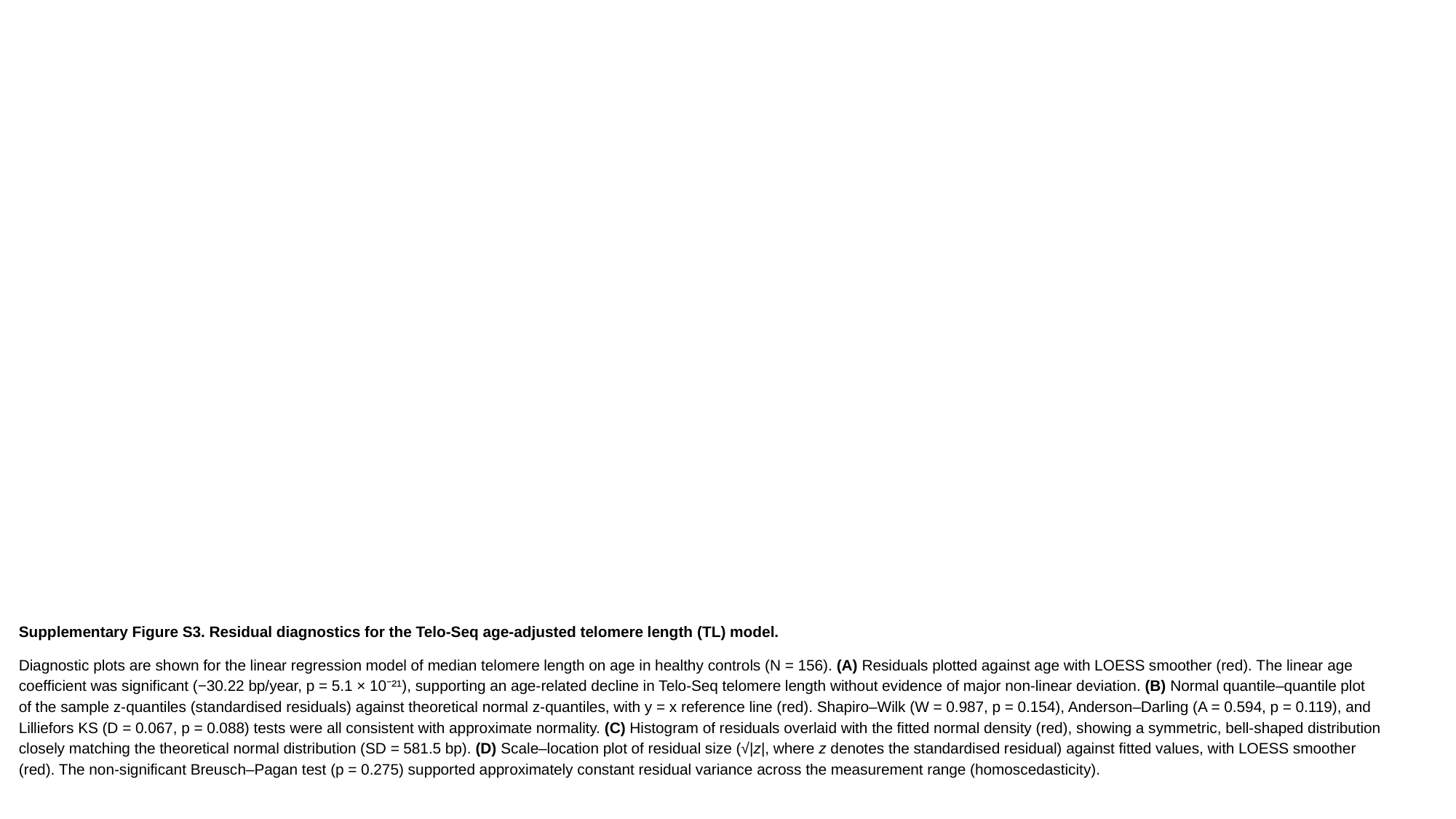

Supplementary Figure S3. Residual diagnostics for the Telo-Seq age-adjusted telomere length (TL) model.
Diagnostic plots are shown for the linear regression model of median telomere length on age in healthy controls (N = 156). (A) Residuals plotted against age with LOESS smoother (red). The linear age coefficient was significant (−30.22 bp/year, p = 5.1 × 10⁻²¹), supporting an age-related decline in Telo-Seq telomere length without evidence of major non-linear deviation. (B) Normal quantile–quantile plot of the sample z-quantiles (standardised residuals) against theoretical normal z-quantiles, with y = x reference line (red). Shapiro–Wilk (W = 0.987, p = 0.154), Anderson–Darling (A = 0.594, p = 0.119), and Lilliefors KS (D = 0.067, p = 0.088) tests were all consistent with approximate normality. (C) Histogram of residuals overlaid with the fitted normal density (red), showing a symmetric, bell-shaped distribution closely matching the theoretical normal distribution (SD = 581.5 bp). (D) Scale–location plot of residual size (√|z|, where z denotes the standardised residual) against fitted values, with LOESS smoother (red). The non-significant Breusch–Pagan test (p = 0.275) supported approximately constant residual variance across the measurement range (homoscedasticity).

## Slide 5
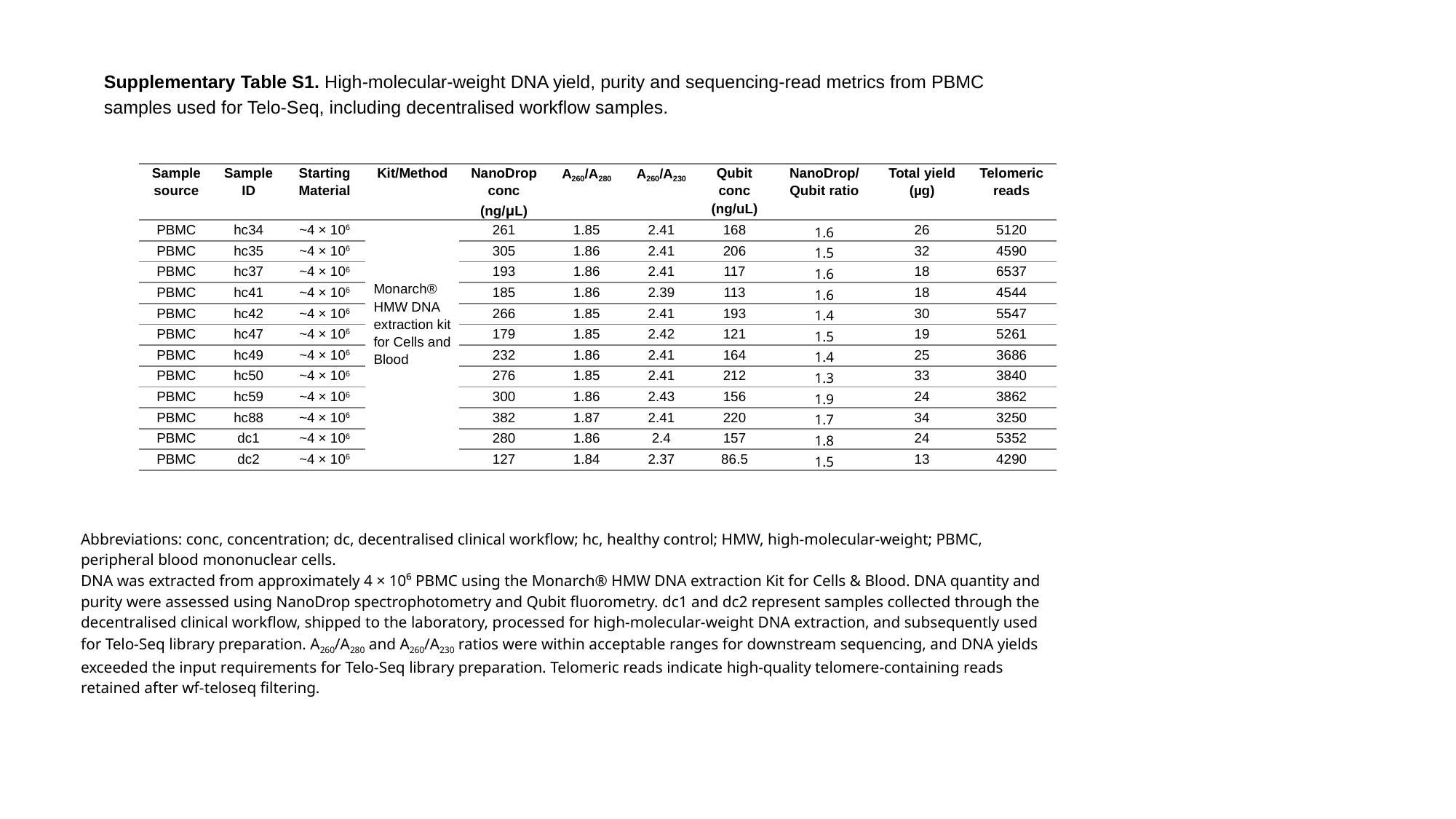

Supplementary Table S1. High-molecular-weight DNA yield, purity and sequencing-read metrics from PBMC samples used for Telo-Seq, including decentralised workflow samples.
| Sample source | Sample ID | Starting Material | Kit/Method | NanoDrop conc (ng/µL) | A260/A280 | A260/A230 | Qubit conc (ng/uL) | NanoDrop/Qubit ratio | Total yield (µg) | Telomeric reads |
| --- | --- | --- | --- | --- | --- | --- | --- | --- | --- | --- |
| PBMC | hc34 | ~4 × 10^6^ | Monarch® HMW DNA extraction kit for Cells and Blood | 261 | 1.85 | 2.41 | 168 | 1.6 | 26 | 5120 |
| PBMC | hc35 | ~4 × 10^6^ | | 305 | 1.86 | 2.41 | 206 | 1.5 | 32 | 4590 |
| PBMC | hc37 | ~4 × 10^6^ | | 193 | 1.86 | 2.41 | 117 | 1.6 | 18 | 6537 |
| PBMC | hc41 | ~4 × 10^6^ | | 185 | 1.86 | 2.39 | 113 | 1.6 | 18 | 4544 |
| PBMC | hc42 | ~4 × 10^6^ | | 266 | 1.85 | 2.41 | 193 | 1.4 | 30 | 5547 |
| PBMC | hc47 | ~4 × 10^6^ | | 179 | 1.85 | 2.42 | 121 | 1.5 | 19 | 5261 |
| PBMC | hc49 | ~4 × 10^6^ | | 232 | 1.86 | 2.41 | 164 | 1.4 | 25 | 3686 |
| PBMC | hc50 | ~4 × 10^6^ | | 276 | 1.85 | 2.41 | 212 | 1.3 | 33 | 3840 |
| PBMC | hc59 | ~4 × 10^6^ | | 300 | 1.86 | 2.43 | 156 | 1.9 | 24 | 3862 |
| PBMC | hc88 | ~4 × 10^6^ | | 382 | 1.87 | 2.41 | 220 | 1.7 | 34 | 3250 |
| PBMC | dc1 | ~4 × 10^6^ | | 280 | 1.86 | 2.4 | 157 | 1.8 | 24 | 5352 |
| PBMC | dc2 | ~4 × 10^6^ | | 127 | 1.84 | 2.37 | 86.5 | 1.5 | 13 | 4290 |
Abbreviations: conc, concentration; dc, decentralised clinical workflow; hc, healthy control; HMW, high-molecular-weight; PBMC, peripheral blood mononuclear cells.
DNA was extracted from approximately 4 × 10⁶ PBMC using the Monarch® HMW DNA extraction Kit for Cells & Blood. DNA quantity and purity were assessed using NanoDrop spectrophotometry and Qubit fluorometry. dc1 and dc2 represent samples collected through the decentralised clinical workflow, shipped to the laboratory, processed for high-molecular-weight DNA extraction, and subsequently used for Telo-Seq library preparation. A260/A280 and A260/A230 ratios were within acceptable ranges for downstream sequencing, and DNA yields exceeded the input requirements for Telo-Seq library preparation. Telomeric reads indicate high-quality telomere-containing reads retained after wf-teloseq filtering.

## Slide 6
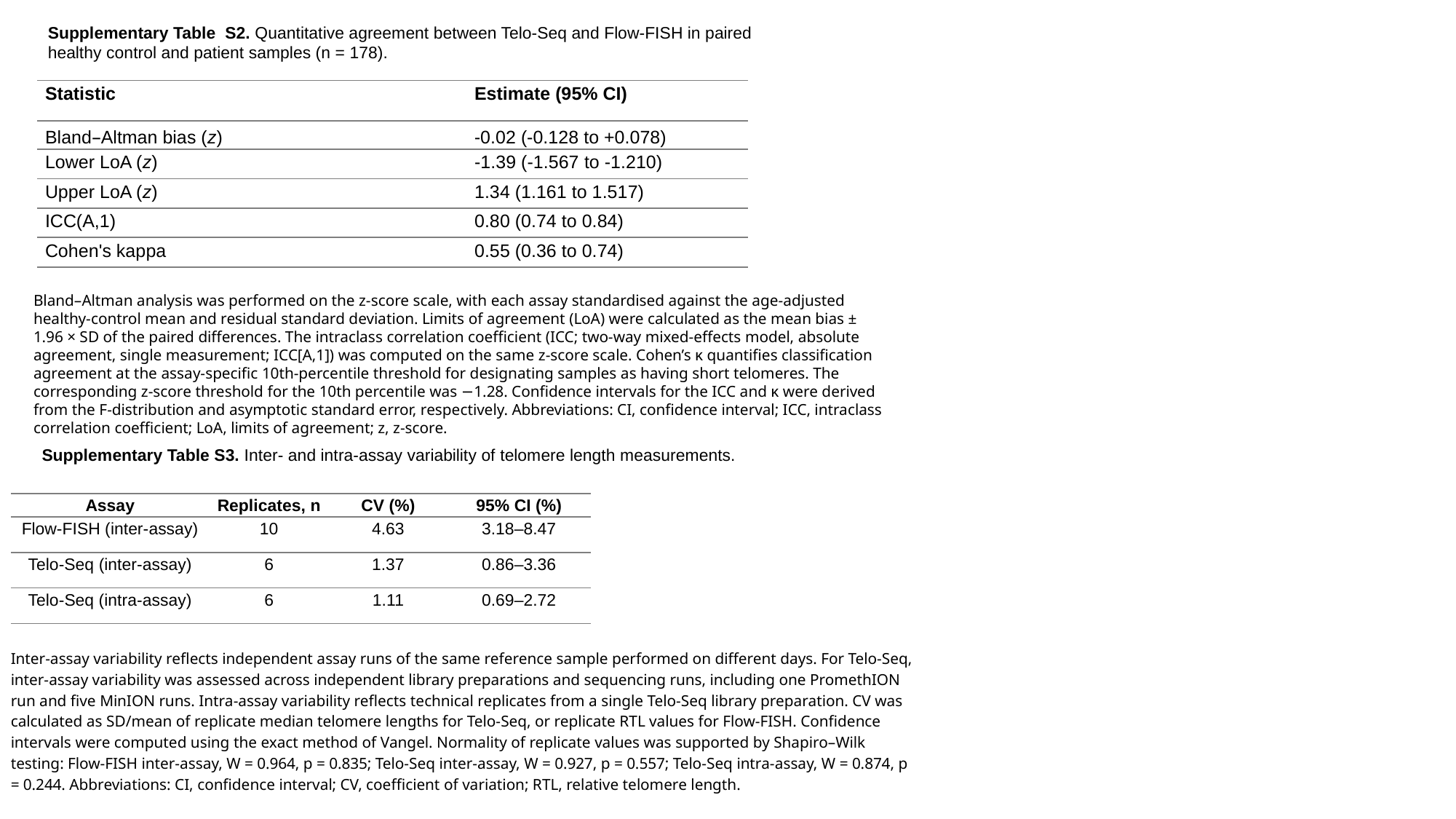

Supplementary Table S2. Quantitative agreement between Telo-Seq and Flow-FISH in paired healthy control and patient samples (n = 178).
| Statistic | Estimate (95% CI) |
| --- | --- |
| Bland–Altman bias (z) | -0.02 (-0.128 to +0.078) |
| Lower LoA (z) | -1.39 (-1.567 to -1.210) |
| Upper LoA (z) | 1.34 (1.161 to 1.517) |
| ICC(A,1) | 0.80 (0.74 to 0.84) |
| Cohen's kappa | 0.55 (0.36 to 0.74) |
Bland–Altman analysis was performed on the z-score scale, with each assay standardised against the age-adjusted healthy-control mean and residual standard deviation. Limits of agreement (LoA) were calculated as the mean bias ± 1.96 × SD of the paired differences. The intraclass correlation coefficient (ICC; two-way mixed-effects model, absolute agreement, single measurement; ICC[A,1]) was computed on the same z-score scale. Cohen’s κ quantifies classification agreement at the assay-specific 10th-percentile threshold for designating samples as having short telomeres. The corresponding z-score threshold for the 10th percentile was −1.28. Confidence intervals for the ICC and κ were derived from the F-distribution and asymptotic standard error, respectively. Abbreviations: CI, confidence interval; ICC, intraclass correlation coefficient; LoA, limits of agreement; z, z-score.
Supplementary Table S3. Inter- and intra-assay variability of telomere length measurements.
| Assay | Replicates, n | CV (%) | 95% CI (%) |
| --- | --- | --- | --- |
| Flow-FISH (inter‑assay) | 10 | 4.63 | 3.18–8.47 |
| Telo-Seq (inter‑assay) | 6 | 1.37 | 0.86–3.36 |
| Telo-Seq (intra‑assay) | 6 | 1.11 | 0.69–2.72 |
Inter-assay variability reflects independent assay runs of the same reference sample performed on different days. For Telo-Seq, inter-assay variability was assessed across independent library preparations and sequencing runs, including one PromethION run and five MinION runs. Intra-assay variability reflects technical replicates from a single Telo-Seq library preparation. CV was calculated as SD/mean of replicate median telomere lengths for Telo-Seq, or replicate RTL values for Flow-FISH. Confidence intervals were computed using the exact method of Vangel. Normality of replicate values was supported by Shapiro–Wilk testing: Flow-FISH inter-assay, W = 0.964, p = 0.835; Telo-Seq inter-assay, W = 0.927, p = 0.557; Telo-Seq intra-assay, W = 0.874, p = 0.244. Abbreviations: CI, confidence interval; CV, coefficient of variation; RTL, relative telomere length.

## Slide 7
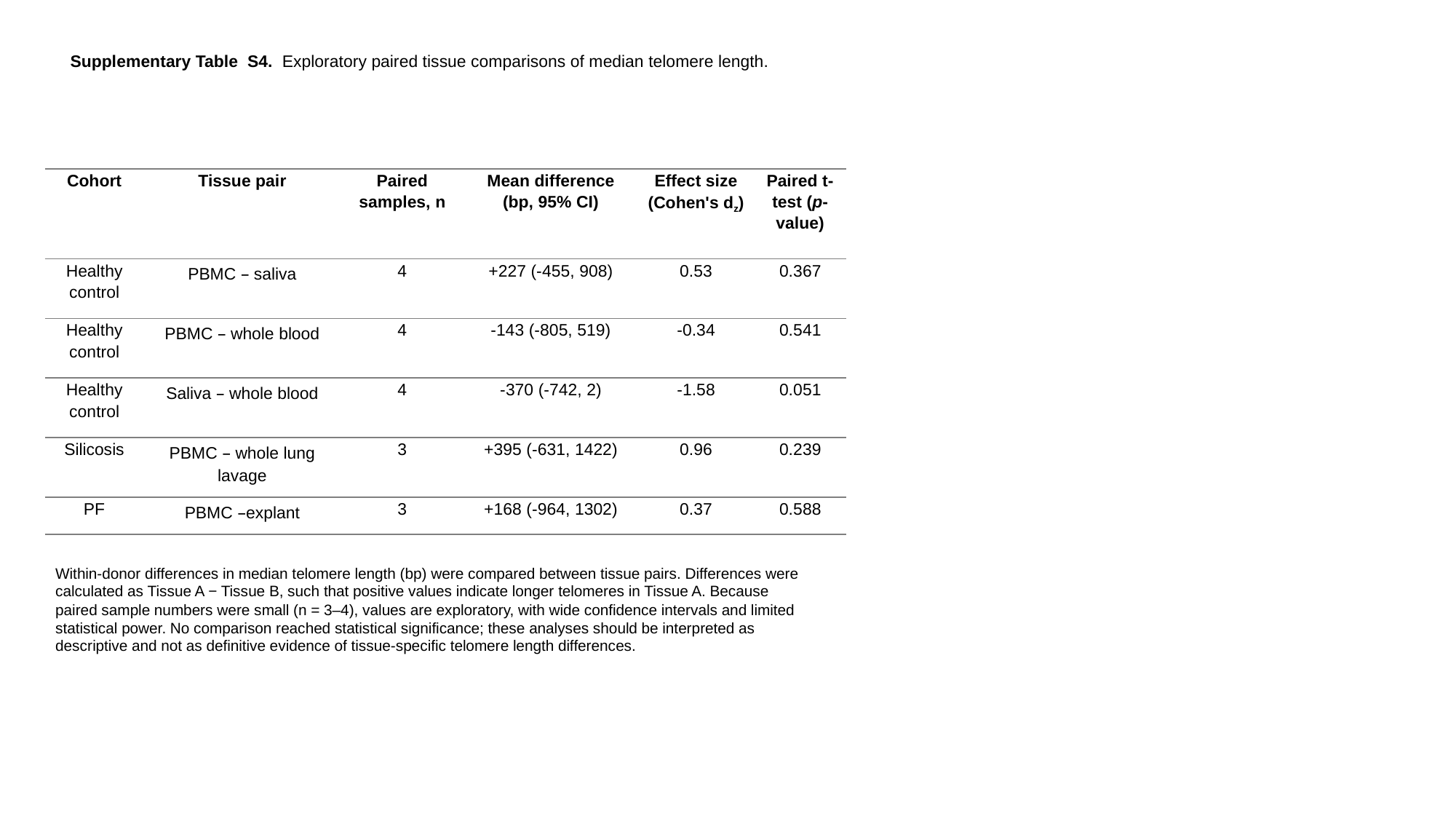

Supplementary Table S4. Exploratory paired tissue comparisons of median telomere length.
| Cohort | Tissue pair | Paired samples, n | Mean difference (bp, 95% CI) | Effect size (Cohen's dz) | Paired t-test (p-value) |
| --- | --- | --- | --- | --- | --- |
| Healthy control | PBMC – saliva | 4 | +227 (-455, 908) | 0.53 | 0.367 |
| Healthy control | PBMC – whole blood | 4 | -143 (-805, 519) | -0.34 | 0.541 |
| Healthy control | Saliva – whole blood | 4 | -370 (-742, 2) | -1.58 | 0.051 |
| Silicosis | PBMC – whole lung lavage | 3 | +395 (-631, 1422) | 0.96 | 0.239 |
| PF | PBMC –explant | 3 | +168 (-964, 1302) | 0.37 | 0.588 |
Within-donor differences in median telomere length (bp) were compared between tissue pairs. Differences were calculated as Tissue A − Tissue B, such that positive values indicate longer telomeres in Tissue A. Because paired sample numbers were small (n = 3–4), values are exploratory, with wide confidence intervals and limited statistical power. No comparison reached statistical significance; these analyses should be interpreted as descriptive and not as definitive evidence of tissue-specific telomere length differences.
